# The role of Pcdh10 in neurological disease and cancer

**DOI:** 10.1007/s00432-023-04743-w

**Published:** 2023-04-14

**Authors:** Yilan Zhen, Macarena Pavez, Xinying Li

**Affiliations:** 1 Menzies Institute for Medical Research, University of Tasmania, Liverpool street, Hobart, 7000 Australia; 2 Department of Anatomy, University of Otago, Dunedin, Otago New Zealand; 3 Department of Oncology, The First Affiliated Hospital of Anhui Medical University, Hefei, People’s Republic of China; 4 School of Life Sciences, Anhui Medical University, Hefei, People’s Republic of China

**Keywords:** Protocadherin10, Neurological disease, Tumor, Cancer, Methylation, Tumor suppressor, Epigenetic therapy

## Abstract

**Background:**

Protocadherin 10 (PCDH 10), a member of the superfamily of protocadherins, is a Ca2^+^-dependent homophilic cell-cell adhesion molecule expressed on the surface of cell membranes. Protocadherin 10 plays a critical role in the central nervous system including in cell adhesion, formation and maintenance of neural circuits and synapses, regulation of actin assembly, cognitive function and tumor suppression. Additionally, Pcdh10 can serve as a non-invasive diagnostic and prognostic indicator for various cancers.

**Methods:**

This paper collects and reviews relevant literature in Pubmed.

**Conclusion:**

This review describes the latest research understanding the role of Pcdh10 in neurological disease and human cancer, highlighting the importance of scrutinizing its properties for the development of targeted therapies and identifying a need for further research to explore Pcdh10 functions in other pathways, cell types and human pathologies.

## Introduction

Cell–cell adhesion is a basic process in the morphogenesis of multicellular organisms. Originally described as cell adhesion molecules, cadherins play a crucial role in cell recognition, cell communication, morphogenesis, cytoskeletal organization, cell migration, and neural circuit formation (Flaherty and Maniatis 2020; Pancho et al. 2020). According to sequence similarities, cadherins can be divided into three subfamilies: classical cadherins, desmosomal cadherins, and protocadherins (Pcdhs) (Halbleib and Nelson 2006). Among these, Pcdhs are the largest and most diverse cadherin subfamily. Pcdhs are highly abundant in the developing brain, lungs and kidneys (Homayouni et al. 2001; Kim et al. 2007), being crucial for organ development and maintenance. Pcdhs are also involved in the establishment and function of specific cell–cell connections as well as in tumor development (Kahr et al. 2013). Based on the genomic organization, Pcdhs are further classified as clustered or non-clustered (Pancho et al. 2020).

Pcdh10 is a non-clustered Pcdh (Light and Jontes 2017) that is initially highly expressed in CNS and is essential for neuronal development (Uemura et al. 2007). Pcdh10 has been identified as an autism-spectrum disorder gene (Morrow et al. 2008; Ferri et al. 2021; Hoshina et al. 2022). Additionally, Pcdh10 is a newly discovered tumor suppressor gene which is downregulated by hypermethylation or genetic deletion in various malignant tumors, and is linked to the occurrence, proliferation, invasion and metastasis of tumors (Zhong et al. 2013; Qiu et al. 2016; Yang et al. 2016, 2022). Importantly, tumor-associated *Pcdh10* methylation status exhibit diagnostic and prognostic value for multiple human cancers, such as colorectal cancer, prostate cancer, cervial cancer, breast cancer, etc. (Lin et al. 2011; Jao et al. 2014; Deng et al. 2016; Liu et al. 2018b). Pcdh10 methylation does not occur in healthy tissues. However, the research of Pcdh10 is still in its early days, and there are many unknown biological characteristics and functions that have not yet been discovered.

In this review, we explore the role of Pcdh10 in neurological disease and human cancer, and provide further insight into the molecular mechanisms and disease-relationship that Pcdh10 controls.

## The biological features of Pcdh10

Non-clustered PCDHs can be classified into three groups: δ1, δ2 and ε subgroup based on their structure and function (Kim et al. 2011). Most non-clustered Pcdhs have 6 or 7 extracellular cadherin repeats in the ectodomain, a transmembrane, and a cytoplasmic domain. Both Pcdhδ1 and Pcdhδ2 members contain conserved cytoplasmic motifs (CM1 and CM2) in their cytoplasmic domain, while Pcdhδ1 members have an additional protein phosphatase-1α binding domain (RRVTE, CM3).

**Fig. 1:**
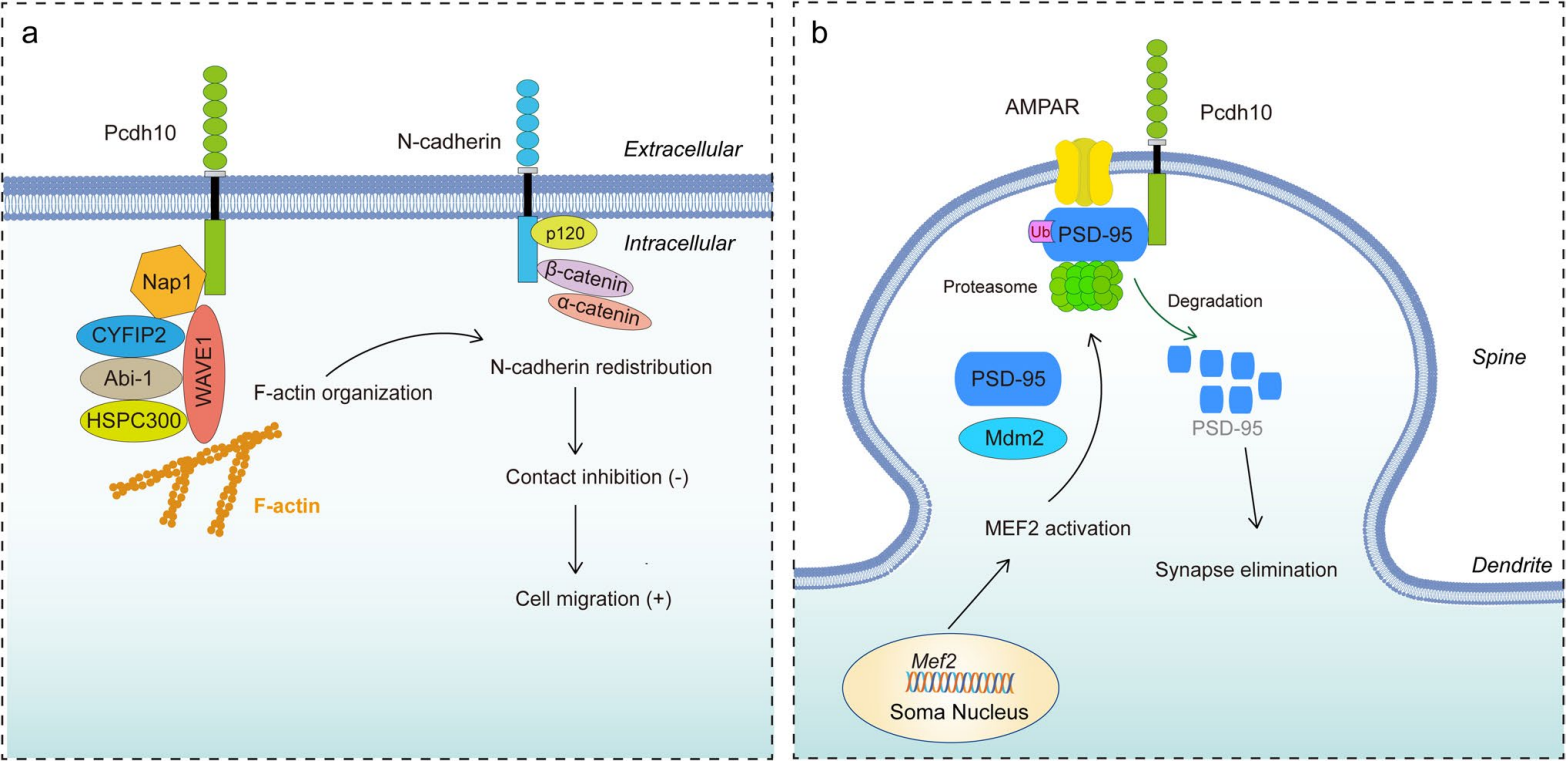
Mechanisms of Pcdh10 activity required to drive and maintain physiological and brain developmental functions. **A** Pcdh10 can bind Nap1, CYFIP2, Abi-1, HSPC300 and WAVE1 to form a Pcdh10-WAVE regulatory complex. By recruiting the WAVE regulatory complex to inter-axonal contact sites, Pcdh10 regulates F-actin organization and N-cadherin redistribution. Redistributed N-cadherin is unable to induce contact inhibition, leading to increased cell migration of glioblastoma cells. **B** Nuclear MEF2 activation initiates Mdm2 transcription, which results in ubiquitination of PSD-95. Pcdh10 then binds to ubiquitinated PSD-95 and links it to the proteasome for degradation, resulting in synapse elimination

Pcdh10, originally named OL-protocadherin, belongs to Pcdhδ2 and is located on chromosome 4 in humans and chromosome 3 in mice. It contains 6 extracellular cadherin repeats in the ectodomain, a transmembrane domain and a unique cytoplasmic domain (Hirano et al. 1999; Kim et al. 2011). Similar to other members of non-clustered Pcdhs, Pcdh10 mediates calcium-dependent cell–cell adhesion by homophilic binding through the extracellular cadherin domains, although this binding ability is generally weak (Hirano et al. 1999). Since cytoplasmic domains of non-clustered Pcdhs are distinct, they can act as major regulators via interacting with a variety of intracellular binding partners (Kim et al. 2011). A short isoform and a long isoform of Pcdh10 have been identified in human. Pcdh10 in mice contains a short isoform (iso1) and three long isoforms (iso2, iso3 and iso4), all of which only different at their carboxyterminal end of cytoplasmic domain (Kleinberger et al. 2022). Interestingly, all three long isoforms of mouse Pcdh10 contain several conserved motifs in their cytoplasmic domains (Kleinberger et al. 2022), suggesting that shared interacting partners are key for the basic functioning of Pcdh10 proteins. For example, mouse Pcdh10 interacts with Nck-associated protein 1 (Nap1), Sra-1/PIR121/cytoplasmic interacting FMR1 protein 2 (CYFIP2), Abl interactor 1 (Abi-1), hematopoietic stem/cell progenitor protein 300 (HSPC300) and WAVE1 to generate a Pcdh10-associated WAVE regulatory complex (Nakao et al. 2008). Overexpression of Pcdh10 recruits the WAVE regulatory complex at inter-axonal contact sites, which results in reorganization of F-actin and N-cadherin at these locations, and subsequently regulates cell migration of astrocytoma U251 cells (Fig. 1a). However, how Pcdh10/ Nap1/WAVE1 complex affect actin assembly needs to be further clarified.

## PCDH10 in brain development

PCDH10 is detectable in nonneuronal tissue such as heart, kidney, lung and trachea (Wolverton and Lalande 2001), however its predominant expression is in the CNS (Kim et al. 2007), further indicating that Pcdh10 is critical for neural development. A high level of expression of Pcdh10 is detected in the striatum, piriform cortex, and preoptic region of the mouse brain at E13.5, but its expression in the globus pallidus is weak (Uemura et al. 2007). In all brain regions, Pcdh10 plays a key role in axon outgrowth and guidance. It has been shown, for instance, that Pcdh10 knockout mice do not form the cerebral peduncle, corticospinal tract, and striatonigral pathway, while corticofugal axons halting in the ventral telencephalon and thalamocortical axons fail to reach the internal capsule (Uemura et al. 2007). During embryonic and postnatal development, Pcdh10 is also expressed in the olfactory system, and variable levels of Pcdh10 are detected in olfactory sensory neurons and the diverse olfactory bulb glomeruli (Aoki et al. 2003; Williams et al. 2011). Pcdh10 expression is activity-dependent within olfactory sensory neurons, as reducing sensory odorant-evoked activity by naris occlusion or expression of an inactive form of cyclic nucleotide gated A2, reduces the expression of Pcdh10. Circuit formation is highly dependent on this regulation of expression, as the misexpression of Pcdh10 significantly impairs glomeruli formation in the olfactory system (Williams et al. 2011).

Neurons of the lateral and basolateral amygdala in mice heterozygous for Pcdh10 also have more filopodia and dendritic spines and (Schoch et al. 2017), and Pcdh10 appears to be necessary for the elimination of hippocampal and cortical synapses (Tsai et al. 2012). Pcdh10 acts as the downstream molecule of myocyte enhancer factor 2 (MEF2), which initiates the transcription of Murine double minute 2 (Mdm2). Post-synaptic scaffolding protein 95 (PSD-95), the synaptic scaffolding protein, was ubiquitinated by Mdm2 in response to MEF2 activation. Pcdh10 then binds PSD-95 and links it to the proteasome for ubiquitination and degradation, leading to synapse elimination (Fig. 1b). These data imply that Pcdh10 is an important player in dendritogenesis, axon development and synaptogenesis.

## Pcdh10 in neurological disease

Autism spectrum disorders (ASD, also known as autism) is a highly genetically heterogeneous neurodevelopmental disorder characterized by impaired social communications. ASD commonly co-presents with other neurological conditions, such as epilepsy, intellectual disability or bipolar disorders. Several studies have identified *Pcdh10* act as an autism associated gene (Morrow et al. 2008; Bucan et al. 2009).

It has been reported that the pathophysiology of autism is highly related with homozygous deletion of Pcdh10 in families with ASD (Morrow et al. 2008). Patients with homozygous Pcdh10 deletion exhibit disrupted elimination of activity-dependent excitatory synapses, as a result of altered ubiquitination and degradation pathways (Tsai et al. 2012). In line with the study, Hoshina and colleague showed that *Pcdh10* deletion in mice display mild impairment in their social recognition and communication responses, suggesting that Pcdh10 mutations may cause ASD-related symptoms (Hoshina et al. 2022).

Heterozygous male mice of this mutant strain display sociability defects (Schoch et al. 2017) and altered γ oscillations (Port et al. 2017), which are known to be crucial for fear memory retrieval (Bocchio et al. 2017). Accordingly, a recent study showed that juvenile and adult Pcdh10-heterozygous mice displayed an increase in immature dendritic spine density, reduced NMDAR expression, altered γ synchronization of the basolateral amygdala, and disrupted fear conditioning behaviours (Ferri et al. 2021). Interestingly, Pcdh10^+/–^ females showed deficits only as adults in the cued fear memory, which might relate to hormonal changes. However, the mechanism underlying the Pcdh10 knockdown-induced behavioral differences between sexes is unclear.

In addition to ASD, several recent studies have implicated human *Pcdh10* in other neurological conditions, such as familial amyloidotic polyneuropathy (FAP), obsessive–compulsive disorder (OCD), major depression (MD) and schizophrenia (Fromer et al. 2014; Goncalves et al. 2016; Qin et al. 2016; Bodea et al. 2017; Tang et al. 2019). A large-scale single nucleotide polymorphism genotyping data on chromosome 4 suggested that *Pcdh10* is one of the susceptibility genes of schizophrenia and bipolar disorder (Tang et al. 2019). Similarly, 428 methylated genes, including Pcdh10, have been linked to early-onset major depression in an epigenome-wide association study of 75 monozygotic twin pairs (Roberson-Nay et al. 2020). As aforementioned, deletion of *Pcdh10* in mice does not affect the growth of striatal and nigrostriatal axons, but rather leads to defects in development of excitatory synapses in the dorsal basolateral nucleus of the amygdala, reduces anxiety, and causes fear and stress in ASD, OCD and MD (Hoshina et al. 2022). These results imply a strong association between Pcdh10 and relevant psychiatric disorders, including ASD, OCD and MD, and also suggest Pcdh10 as a potential target for designing anxiolytics.

## Role of Pcdh10 in cancers

Numerous studies have reported that Pcdh10 acts as a tumor suppressor in a wide range of human tumors. Although the *Pcdh10* gene is widely expressed in normal tissues, it is silenced or decreased in malignant tumors.

### Pcdh10 in colorectal cancer

Colorectal cancer (CRC) is one of the most common types of malignant tumors. Pcdh10 is well-known as a tumor suppressor in colorectal carcinogenesis, invasion and metastasis (Zhong et al. 2017).

Several studies have reported that aberrant CpG methylation of the Pcdh10 promoter is observed in 43–85% of colorectal cancer tissues, indicating that downregulated Pcdh10 caused by methylation is a common feature of colorectal carcinogenesis (Yu et al. 2010; Silva et al. 2013; Zhong et al. 2013). A recent microarray analysis has reported that Pcdh10 expression is lost in more than half of patients with CRC (Skuja et al. 2019), raising the possibility that genetic deletion could be another mechanism for Pcdh10 inactivation in CRC. Mutations in BRAF genes, which are linked to dysregulated DNA methylation (Tanaka et al. 2010), has been found in about 10% of CRC cases (Caputo et al. 2019). Dobre and colleagues further demonstrated *BRAF* positive cases have higher Pcdh10 methylation levels than *BRAF* negative cases (Dobre et al. 2021). Taken together, Pcdh10 genetic modification and epigenetic inactivation play critical roles in the development of CRC.

In support of a role for Pcdh10 as a potential antioncogene, re-expressing Pcdh10 in colorectal cancer RKO cells leads to G1 cell cycle arrest without affecting apoptosis (Zhong et al. 2013). More specifically, Pcdh10 could inhibit cell proliferation and survival by modulating p53/p21/Rb and Bcl-2 pathways in CRC cells (Jao et al. 2021). Meanwhile, it also suppressed epithelial‐mesenchymal transition (EMT), a cellular biological process that promotes cancer cells to migrate, and stemness in CRC by negatively affecting the EGFR/AKT/GSK3β/β‐catenin signaling pathway (Jao et al. 2021).

In addition to tumor inhibition, methylation of Pcdh10 may serve as a non-invasive biomarker for CRC diagnosis as Pcdh10 methylation that is present in tissues could be detected in serum/plasma (Danese et al. 2013). Pcdh10 methylation detected in plasma increases with increasing methylation rate in tumor tissues only in early CRC (stage I/II). Additionally, allelic loss of Pcdh10 was ascertained in primary CRC tumors, and highly related with tumor progression and distant metastasis, suggesting that its allelic loss predicts an adverse prognosis (Jao et al. 2014). Moreover, patients receiving adjuvant treatment with no methylation in Pcdh10, SPARC and UCHL1, had longer disease-free rates and overall survival rates than those with hypermethylation (Heitzer et al. 2014). In contrast, unmethylated genes were related to shorter survival in surveillance group. These findings suggest that promoter methylation status of *Pcdh10, SPARC* and *UCHL1* provide a suitable tool for predicting prognosis of stage II colorectal cancer patients.

### Pcdh10 in tumors of the female reproductive system

Tumors in the female genital tract represent a leading cause of morbidity and mortality among women worldwide. Cervical and endometrial cancers are two very different diseases, having differing pathogenesis and treatments. However, PCDH10 promoter hypermethylation is a frequent hallmark observed during the progression of cervical and endometrial cancers, as previously reported (Narayan et al. 2009; Wang et al. 2009; Zhao et al. 2014; Bhat et al. 2017).

According to GEO2R analysis, Pcdh10 is downregulated and is likely to be one of the most significant genes in tumor differentiation in endometrial cancer (Liu et al. 2018a). Endometrial cancer is the most common gynecologic malignant cancer and about 80% of these cancers are endometrial endometrioid carcinomas (EEC). Pcdh10 is repressed in EEC cells due to its promoter CpG hypermethylation. A novel PCDH10-Wnt/β-catenin-MALAT1 regulatory axis that contributes to ECC development and progression, delays tumor growth and induces cell apoptosis (Zhao et al. 2019). Yang and colleagues also identified DEPDC1 as a downstream mediator of Pcdh10, and they further demonstrated that Pcdh10 suppress cell proliferation and induce apoptosis through DEPDC1-caspase signaling in EEC cell lines (HEC-1-A and KLE) (Yang et al. 2016). In the future, it would be interesting to investigate the clinical significance of Pcdh10 and MALAT1/DEPDC1. Moreover, a recent study has reported that low expression of Pcdh10 is associated with high Enhancer of Zeste Homolog 2 (EZH2) expression and Histone H3 (H3K27me3) enrichment in the tissue of endometriosis patients (Xiaolei et al. 2022). Silencing EZH2 by siRNA reduced H3K27me3 enrichment and increased PCDH10 expression, resulting in decreased invasion and migration of endometrial stromal cells and providing a target for the treatment of endometriosis patients (Xiaolei et al. 2022).

Similarly, Pcdh10 is also inactivated epigenetically in 75% cervical cell lines (Ying et al. 2006). In cervical Hela cells, knockdown of HOTAIR lncRNAs inhibits the Wnt/β-catenin signaling cascade by decreasing promoter methylation of Pcdh10, demonstrating the potential mechanism of how Pcdh10 reguates the progression of cervical cancer (Salmeron-Barcenas et al. 2019). Notably, analysis of Pcdh10 in cervical scrapings is superior to the Human Papillomavirus (HPV) test, implying its potential function as a specific diagnostic biomarker (Lin et al. 2011). Collectively, these findings demonstrate the potential role of Pcdh10 in inducing the development of different tumors of the female genital tract.

### Pcdh10 in gastric cancer

Gastric cancer (GC) is the third most common fatal form of cancer around the globe and the detailed mechanism underlying gastric carcinogenesis remains unclear. Pcdh10 expression is silenced or down-regulated in gastric cancer cells and tissues (Yu et al. 2009; Li et al. 2012b), suggesting it may act as a tumor suppressor in GC. Re-expression Pcdh10 in MKN45 gastric cancer cells inhibited tumor growth, cell proliferation and invasion, induced cell apoptosis, and also increased the expression of pro-apoptotic genes including Fas, Caspase8, Jun, and CDKN1A; the anti-proliferation gene FGFR; and the anti-invasion gene HTATIP2 (Yu et al. 2009). Another study showed Pcdh10 overexpression in gastric cancer cell lines (MNK74, 7901 and AGS) suppressed cell proliferation but had no effect on cell apoptosis (Li et al. 2012b). Further investigations are required to fully understand the function of Pcdh10 in regulating apoptosis in gastric cancer.

Numerous studies have indicated that aberrant methylation of Pcdh10 could be used as a non-invasive biomarker to facilitate diagnosis and prognostic guidance for gastric cancer patients (Deng et al. 2014; Hou et al. 2015; Schneider et al. 2015; Pimson et al. 2016). For example, using MSP qPCR method, Pimson and colleagues demonstrated that Pcdh10 promoter methylation was detected in 94.06% of plasma DNA from gastric cancer patients whereas it was found in only 2.97% of matched controls, serving as a reliable non-invasive diagnostic indicator for GC (Pimson et al. 2016). In terms of prognosis prediction, Pcdh10 promoter methylation at CpG site was found in 91.92% in GC tissues (Deng et al. 2014). GC patients with 5 or more methylated CpG sites of PCDH10 promoter were dramatically related to poorer survival rates. Meanwhile, using multivariate survival analysis, the authors demonstrated methylation of combined CpG sites (− 115, − 108, − 13, and + 3) was an independent predictor, with overall survival, of gastric cancer patients postoperatively (Deng et al. 2014). Multiple studies have also confirmed this finding (Hou et al. 2015; Schneider et al. 2015). Therefore, Pcdh10 methylated at CpG sites has significant clinical applicability for GC prognosis evaluation.

### Pcdh10 in pancreatic cancer

Pancreatic cancer (PC) is one of the most lethal diseases worldwide (Kamisawa et al. 2016). To date, surgical resection is the best choice for treatment of PC, however, the recurrence rate of patients who undergo resection remains very high (Ilic and Ilic 2016). Therefore, the identification of new predictive biomarkers and exploration of the pathogenesis is crucial for the development of novel therapeutics for management of PC.

Previous study identified that Pcdh10 expression is silenced by methylation in pancreatic cancer cell lines, and re-expression of Pcdh10 prevents the malignant biological process of PC cells (Qiu et al. 2016). An earlier study analyzed Pcdh10 promoter methylation in pancreatic tumor samples, but high-resolution melting analysis failed to detect a significant association between Pcdh10 promoter methylation status and tumor-staging (Yu et al. 2010). Recently, high methylation levels of Pcdh10 were found to correlate with worse progression-free survival rates instead of the overall survival, suggesting that Pcdh10 methylation status predicts poor prognosis in patients with pancreatic ductal adenocarcinomas (Curia et al. 2019).

In terms of anti-tumor effects, Pcdh10 overexpression can prevent the proliferation, migration, invasion ability of pancreatic cancer cells and trigger apoptosis by activating the AKT pathway (Qiu et al. 2016). Meanwhile, Pcdh10 can interact with human telomerase reverse transcriptase (hTERT) to reduce telomerase activity, hence mediating the inhibitory effect of PC phenotype (Zhou et al. 2015). Zhang and colleagues demonstrated that the Pcdh10 gene could generate circular RNA of Pcdh10 (circPcdh10) in PC tissue, indicating a worse prognosis (Zhang et al. 2021).

### Pcdh10 in other cancers

The deletion of Pcdh10 has been reported in various human tumors. In addition to the aforementioned CRC, GC, PC, cervical and endometrial cancers, Pcdh10 loss has been observed in non-small-cell lung cancer (NSCLC; (Tang et al. 2012), nasopharyngeal and esophageal cancer (Ying et al. 2006), bladder cancer (Lin et al. 2012, 2013), hepatocellular carcinoma (Fang et al. 2013; Bing et al. 2018), multiple myeloma (Li et al. 2012a), lymphoid malignancies (Narayan et al. 2013), medulloblastoma (Bertrand et al. 2011), breast cancer (Liu et al. 2018b), and prostate cancer (Li et al. 2011), implying that Pcdh10 plays an oncosuppressor role in tumors.

In support of a role for Pcdh10 as a tumor suppressor, restoration of Pcdh10 in hepatocellular carcinoma cell lines inhibits proliferation and induces cell apoptosis via suppressing PI3K/Akt signaling pathway (Ye et al. 2017). In multiple myeloma cells, rescue of Pcdh10 expression induces apoptosis by impeding the the NF-κB pathway (Li et al. 2014), and suppresses cell proliferation via the negative modulation of Wnt/β-catenin/BCL-9 signaling (Xu et al. 2015). In oncogenic KRAS-mutated NSCLC mouse model, KRAS mutation increases the expression of Miz1, which in turn suppresses Pcdh10, leading to enhanced cell proliferation and promotion of lung tumorigenesis (Yang et al. 2022). Further evidence that Pcdh10 acts as an oncosuppressor derives from the observations that downregulated Pcdh10 expression caused by methylation predicted poor prognosis in patients with hepatocellular carcinoma (Bing et al. 2018), breast cancer (Liu et al. 2018b; Xu et al. 2021), prostate cancer (Wang et al. 2014; Deng et al. 2016), and non-small-cell lung cancer (Harada et al. 2015). These data also indicated that Pcdh10 methylation was a potential prognostic biomarker for those human cancers.

It should be noted that Pcdh10 might act as a tumor oncogene in gliomas, as it is essential for the proliferation and tumorigenicity of human glioblastoma cell lines GB2 and GB16 (Echizen et al. 2014). In human astrocytoma cell (U251), the cytoplasmic domain of Pcdh10 can interact with Nap1 and recruit the WAVE complex, and this interaction promotes adhesion and motility at the cell junctions to facilitate migration (Nakao et al. 2008). However, Pcdh10 signaling has the opposite effect in medulloblastoma cells, where Pcdh10 expression is decreased due to DNA hypermethylation and histone modification, but its restoration impedes migration (Bertrand et al. 2011). Similarly, after treatment with cytochalasin H in U87MG malignant human glioma cells, the proliferation is inhibited along with upregulated Pcdh10 expression (Heidarzadeh et al. 2019). It is not yet clear how Pcdh10 can promote tumorigenicity under one circumstance and impede it under another.

It is interesting to note that the methylation status of Pcdh10 may be able to predict the response of lymphomas to doxorubicin (Narayan et al. 2013), a common chemotherapeutic drug used to treat a variety of human cancers. Both B-cell (100%) and T-cell (79%) acute lymphoblastic leukemia frequently exhibit Pcdh10 promoter hypermethylation. Non-Hodgkin lymphoma (NHL) cell lines with down-regulated Pcdh10 expression were less sensitive to leukemia specific drugs including dexamathasone and methotrexate, while T-cell and B-cell lymphoma cell lines with Pcdh10 methylation or down-regulated expression showed doxorubicin resistance, providing new evidence for the selection of treatment plans (Narayan et al. 2013). Meanwhile, Pcdh10 could be a potential target gene for establishing epigenetic therapies in lymphomas. Imatinib is a molecular target drug used to treat chronic myeloid leukemia. In imatinib-resistant K562 leukemia cell line (KR cells), silencing of hBEX1 can repress imatinib-induced apoptosis (Ding et al. 2009). Gain expression of hBex1 enhanced PCDH10 expression and partially restored sensitivity to imatinib, implying a novel hBex1/PCDH10 pathway which contributes to drug resistance. However, the mechanism of the involvement of Pcdh10 in apoptosis has not been examined (Table 1).

**Table 1:** Functions of Pcdh10 in various cancer

Disease	Expression	Property	Genes/Proteins/Pathways	Function	References
Colorectal cancer	Down	Antioncogene	p53/p21/Rb, Bcl-2, EGFR/AKT/GSK3β/β‐catenin signaling pathway	Proliferation, apoptosis, EMT, stemness	Jao et al. (2021)
Endometrial cancer	Down	Antioncogene	MALAT1, wnt/β-catenin signaling pathway	Tumor growth, apoptosis	Zhao et al. (2019)
	Down	Antioncogene	DEPDC1	Proliferation, apoptosis	Yang et al. (2016)
	Down	Antioncogene	EZH2, H3K27me3	Migration, invasion	Xiaolei et al. (2022)
Gastric cancer	Down	Antioncogene	Fas, Caspase8, Jun, CDKN1A, FGFR2, HTATIP2	Tumor growth, apoptosis, invasion, metastasis	Yu et al. (2009)
	Down	Antioncogene	Unknown	Proliferation	Li et al. (2012a, b)
Pancreatic cancer	Down	Antioncogene	PARP, caspase-3, caspase-9, Bcl-2; Akt signaling pathway	Proliferation, migration, invasion, Apoptosis	Qiu et al. (2016)
	Down	Antioncogene	hTERT	Proliferation, migration, invasion	Zhou et al. (2015)
Hepatocellular carcinoma	Down	Antioncogene	PI3K/Akt signaling pathway	Proliferation, apoptosis	Ye et al. (2017)
Multiple myeloma	Down	Antioncogene	NF-κB pathway	Apoptosis	Li et al. (2014)
	Down	Antioncogene	Wnt/β-catenin/BCL-9 signaling pathway	Proliferation	Xu et al. (2015)
KRAS-mutated non-small-cell lung cancer	Down	Antioncogene	MiZ1	Proliferation, tumor growth	Yang et al. (2022)
Glioblastoma	Up	Oncogene	Unkown	Proliferation	Echizen et al. (2014)
Medulloblastoma	Down	Antioncogene	Unknown	Migration	Bertrand et al. (2011)
Lymphoid malignancies	Down	Antioncogene	Unknown	Drug resistance	Narayan et al. (2013)
Chronic myeloid leukemia	Down	Antioncogene	hBex1	Drug resistance	Ding et al. (2009)

*EGFR* Epidermal growth factor receptor, *EMT* epithelial-mesenchymal transition, *DEPDC1* DEP domain containing 1, *EZH2* enhancer of Zeste Homolog 2, *CDKN1A* cyclin Dependent Kinase Inhibitor 1A, *FGFR2* fibroblast growth factor receptor 2, *HTATIP2* HIV-1 tat interactive protein 2, *hTERT* human telomerase reverse transcriptase, *Miz1* Myc-interacting zinc-finger protein 1, *hBex1* human brain expressed X-linked 1

## Potential epigenetic therapies targeting Pcdh10

An increasing number of studies have demonstrated that Pcdh10 plays an important role in cancer. Therefore, exploring therapeutic strategies targeting Pcdh10 may be of great importance in the management of several types of tumors.

Therapeutic strategies that target Pcdh10 may be relevant to CRC. Zhou et al. demonstrated that hsa_circ_0001666 functions as a tumor suppressor by directly binding miR‐576‐5p and lessening its inhibitory effect on the target gene Pcdh10, thereby inhibiting cell proliferation, metastasis, EMT progression and stemness as well as triggering apoptosis of CRC cells (Zhou et al. 2021). Notably, hsa_circ_0001666 can also suppress Wnt/β‐catenin signaling, a well‐known cancer‐promoting pathway, via promoting PCDH10 expression.

As previously mentioned, HOTAIR lncRNA acts as the upstream regulator of Pcdh10 in cervical hela cells (Salmeron-Barcenas et al. 2019). The expression of multiple mRNAs, including MAGI2, AJAP1, SOX17, PCDH10, and TET1, was downregulated by HOTAIR knockdown, which also reduced the activity of the Wnt/-catenin signaling pathway. Similarly, HOTAIR interacted with miR-148 and DNMT1, promoting the methylation of PCDH10, and bringing about oncogenic changes in GC (Seo et al. 2021). Moreover, canonical oncogenic lncRNA MALAT1 can bind EZH2 to counteract PCDH10 by inducing the methylation of its promoter, resulting in an increase in GC cell migration and invasion (Qi et al. 2016).

**Fig. 2:**
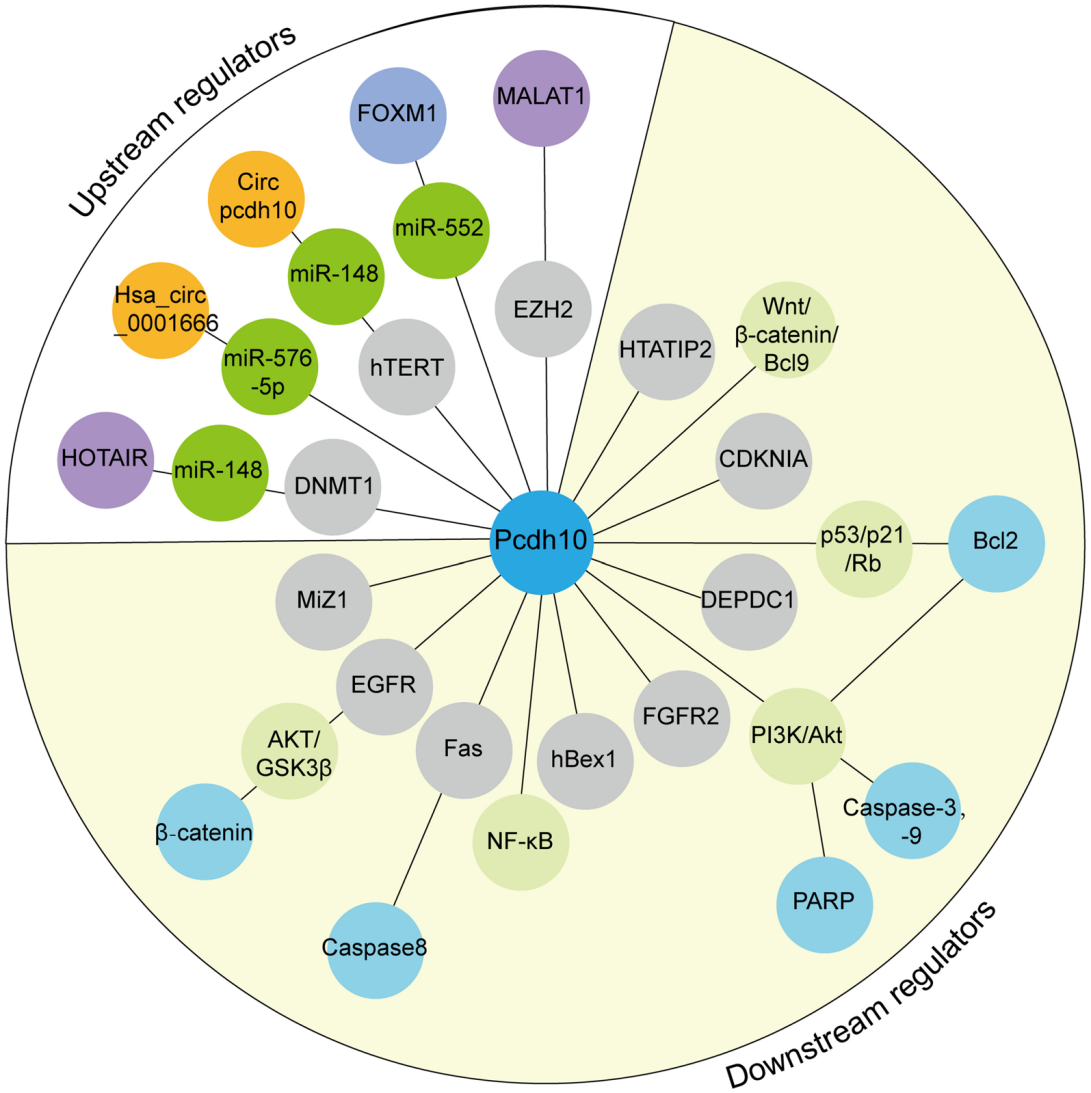
Genes involved in the Pcdh10 regulatory network in human cancer. Genes listed in white background are upstream regulators of Pcdh10, genes listed in light yellow background are downstream regulators of Pcdh10

CircPcdh10 promotes tumor progression of pancreatic cancer by increasing hTERT expression through interacting with miR-338-3p (Zhang et al. 2021). Further experiments confirmed that there was a targeted regulatory association between CircPcdh10 and miR-338-3p/hTERT; the inhibitory effects of circPCDH10 depletion on the viability, proliferation, invasion, and migration of PC cells were significantly abolished by treating with miR-338-3p inhibitor and hTERT. Similarly, a recent study revealed an oncogenic transcription factor FOXM1 which activated expression of miR-552, and further inhibited downstream target genes including Pcdh10, DACH1 and SMAD, which in turn promoted tumor progression and resulted in poor prognosis in PC patients (Wang et al. 2021). However, the in-depth molecular mechanisms underlying these conditions require further elucidation. In general, these results highlight the potentiality of targeting Pcdh10 gene in human cancers (Fig. 2; Table2).

**Table 2:** A summary of lncRNA/miRNAs/circRNA targeting Pcdh10 in cancer

lncRNA/miRNA/circRNA	Cancer type	Functions	Molecule/Pathway	References
Hsa_circ_0001666/miR‐576‐5p	Colorectal cancer	Proliferation, EMT, metastasis, stemness, apoptosis	Wnt/β‐catenin pathway	Zhou et al. (2021)
HOTAIR	Cervial cancer	Unknown	Wnt/β-catenin pathway	Salmeron-Barcenas et al. (2019)
HOTAIR-miR-148	Gastric cancer	Proliferation, apoptosis, invasion, migration	DNMT1	Seo et al. (2021)
MALAT1	Gastric cancer	Migration, invasion	EZH2	Qi et al. (2016)
CircPcdh10/miR-338-3p	Pancreatic cancer	Proliferation, invasion, migration	hTERT	Zhang et al. (2021)
miR-552	Pancreatic cancer	Migration, metastasis	FOXM1	Wang et al. (2021)

*EMT* epithelial-mesenchymal transition, *DNMT1* DNA methyltransferase 1, *EZH2* enhancer of zeste homolog 2, *hTERT* human telomerase reverse transcriptase, *FOXM1* forkhead box protein M1

## Conclusion

The functions of Pcdh10, its regulatory targets and the role it plays in human pathologies, remain largely unexplored. Pcdh10 is considered to play important roles in brain development and is implicated in human neurological disorders like autism, obsessive–compulsive disorder, major depression and schizophrenia. Pcdh10 has also been implicated in a range of human cancers, acting as a tumor suppressor and playing key roles in regulating tumor growth, invasion and metastasis. In contrast, Pcdh10 has also been shown to be an oncogene for the tumorigenesis of glioblastoma. Further research is required to fully elucidate the role of Pcdh10 in neurological conditions and different types of the cancers, and determine whether Pcdh10 is implicated in any other human condition. In addition, aberrant methylations of Pcdh10 have been recognized as a non-invasive biomarker for tumor diagnosis and prognosis. Though the involvement of Pcdh10 in the pathogenesis of neural diseases and human cancers has been recently established, our understanding of the molecular functions and related signaling pathways involved is limited. Moreover, the genes targeting Pcdh10 function and the relevant molecular mechanisms involved also remain to be investigated but could provide further insights into therapeutic strategies that could be developed for the treatment of Pcdh10-regulated conditions. Our review focuses on the known conditions where Pcdh10 is disrupted and its potential as a cancer biomarker, however given the various pathways regulated by Pcdh10, further research is likely to determine its role in other conditions and further explore its potential as a non-invasive biomarker of disease.
